# Evaluating the Economic Impact of Smart Care Platforms: Qualitative and Quantitative Results of a Case Study

**DOI:** 10.2196/medinform.5012

**Published:** 2016-10-31

**Authors:** Frederic Vannieuwenborg, Thomas Van der Auwermeulen, Jan Van Ooteghem, An Jacobs, Sofie Verbrugge, Didier Colle

**Affiliations:** ^1^ Internet Based Communication Networks and Services research group (IBCN) Department of Information Technology (INTEC) Ghent University - iMinds Ghent Belgium; ^2^ Studies on Media, Information & Telecommunication (SMIT) Vrije Universiteit Brussel - iMinds Brussel Belgium

**Keywords:** medical informatics applications, home care services, ambulatory care information systems, evaluation studies as topic, cost-benefit analysis

## Abstract

**Background:**

In response to the increasing pressure of the societal challenge because of a graying society, a gulf of new Information and Communication Technology (ICT) supported care services (eCare) can now be noticed. Their common goal is to increase the quality of care while decreasing its costs. Smart Care Platforms (SCPs), installed in the homes of care-dependent people, foster the interoperability of these services and offer a set of eCare services that are complementary on one platform. These eCare services could not only result in more quality care for care receivers, but they also offer opportunities to care providers to optimize their processes.

**Objective:**

The objective of the study was to identify and describe the expected added values and impacts of integrating SCPs in current home care delivery processes for all actors. In addition, the potential economic impact of SCP deployment is quantified from the perspective of home care organizations.

**Methods:**

Semistructured and informal interviews and focus groups and cocreation workshops with service providers, managers of home care organizations, and formal and informal care providers led to the identification of added values of SCP integration. In a second step, process breakdown analyses of home care provisioning allowed defining the operational impact for home care organization. Impacts on 2 different process steps of providing home care were quantified. After modeling the investment, an economic evaluation compared the business as usual (BAU) scenario versus the integrated SCP scenario.

**Results:**

The added value of SCP integration for all actors involved in home care was identified. Most impacts were qualitative such as increase in peace of mind, better quality of care, strengthened involvement in care provisioning, and more transparent care communication. For home care organizations, integrating SCPs could lead to a decrease of 38% of the current annual expenses for two administrative process steps namely, care rescheduling and the billing for care provisioning.

**Conclusions:**

Although integrating SCP in home care processes could affect both the quality of life of the care receiver and informal care giver, only scarce and weak evidence was found that supports this assumption. In contrast, there exists evidence that indicates the lack of the impact on quality of life of the care receiver while it increases the cost of care provisioning. However, our cost-benefit quantification model shows that integrating SCPs in home care provisioning could lead to a considerable decrease of costs for care administrative tasks. Because of this cost decreasing impact, we believe that the integration of SCPs will be driven by home care organizations instead of the care receivers themselves.

## Introduction

### A Societal Challenge

Many parts of the world face the same social evolution: an aging society. It’s a challenge because with an aging society the demand for care increases while resource availability (both human and monetary) is under pressure.

Information and Communication Technology (ICT)-enabled care services have the potential to reduce costs while maintaining or increasing the quality of care. Many examples in the primary (the general practitioners and health centers) and secondary care sectors (hospitals and specialist) already exist. Electronic health records and electronic drug prescriptions are only a couple of many examples. All these types of ICT-enabled services foster better care communication, organization, less medication or diagnostic errors, and more transparent data sharing [1].

### eCare Services

In recent years, focus intensified on aging in place and how ICT-enabled services could support this. The number of ICT-supported care applications (eCare) such as remote fall detection [2], social contact enhancing applications, and telecare services to diagnose patients remotely [3] grew exponentially. This has resulted in a fragmented and scattered landscape of eCare applications. Most of the services have individual and standalone characteristics but interoperability often lacks [4].

### Smart Care Platforms

In response to this barrier of noninteroperability and nonintegrated eCare services, the introduction of smart care platforms (SCPs) can be witnessed [5-9]. These SCPs allow integration, monitoring, and data exchange between a set of home care applications and services that run on a central cloud-like platform. Smart care platforms foster better care communication and information sharing among the professional, informal care providers, and care receivers [10]; therefore, SCPs are not the same as telecare services though they can support them. Furthermore, many SCPs allow the integration with various monitoring sensors that provide specific context information (eg, room temperature, movement of the person, bed detection, sound level) [11]. Longitudinal analyses of these data give meaningful insights in evolution of the condition of the care receivers and their daily life patterns. In general, the functionalities of SCPs in terms of providing services can be categorized and summarized as follows [9]:

#### Support Care and Care Processes

Examples of these services are: online meal delivery services, alerting specific care actors in case of certain events, care journals, and care agendas.

#### Sharing Care Information and Care Communication

According to the role-based rights of the involved actors (eg, GP vs informal caregiver vs care receivers), one can add, change, erase, or annotate particular information of the care receiver.

#### Support Social Life and Activities

Making video calls with friends or relatives or being able to share some memories with family are just some of these services that support the social life of the home care receiver.

#### Monitoring Services

Integration of various sensors into the homes of the care receivers allows monitoring of context data such as movement, pressure sensors to detect bed or couch presence, accelerometers to detect falls, light, noise, temperature, humidity, smoke detectors, weighing scales, and so on. Through these sensors all kinds of biometric or context information can be captured. Analysis of sensor data allows evaluations of lifestyle trends.

Most SCPs exist with one or more of the above described functionalities. In other cases, SCPs provide the basic set of functionalities, which can easily be extended by adding modular services [12]. O’CareCloudS (OCCS) [12], the SCP developed in the identically named research project is a complete cloud-based platform. The basic service set of OCCS does provide several services to foster better care information sharing and social connectivity. The complete service set covers: (1) consulting and annotating the shared care record, (2) time and task registration of the care givers, (3) care agenda and a smart task list, (4) social calendar, (5) smart messaging service, and (6) a service catalogue for additional OCCS services. In addition, modular lifestyle monitoring services can be added by installing the necessary sensors. Although SCPs can support care provisioning for all types of home care receivers, in this work focus is on elderly, as it can be expected that more elderly will stay longer at home.

### Evaluating Smart Care Platforms

SCPs are believed to have a positive impact on the quality and the cost efficiency of care. But at the same time the main characteristic of SCPs, the ability to connect multiple actors, poses challenges for its adoption. Multi-actor or multi-stakeholders systems require at least a neutral and preferably a positive perceived impact for every actor involved before a successful adoption is possible [13]. Also it’s not clear which actor will initiate the adoption of SCPs.

Therefore, this paper focuses on determining and quantifying the impact of integrating SCPs into present home care processes for the elderly; in other words, evaluating the potential effect or added value of SCPs.

In the literature, previous work on several aspects of the evaluation of SCPs can be found. We distinguish (1) research on the evaluation methodology and (2) results of evaluation processes of eCare services.

The nature of SCPs and eCare services in general requires a multi-aspect evaluation method. Evaluating these services solely based on their medical impact would be insufficient; also focusing on economic impact would be too narrow. Evaluating eCare services in their totality requires looking at them from several angels such as the economic perspective, the medical impact, the social aspect, the impact on the involved actors, legal issues, and technical barriers [13,14].

In this knowledge, different frameworks are developed especially for evaluating eCare services [13-16]. All of them present a model or framework that takes into account several perspectives of the impact of integrating eCare or SCP services. Salvi et al [17] presents an overall evaluation framework based on quality of eCare services in the context of ambient assisted living. This incorporates many quality characteristics such as functionality, reliability, efficiency, and usability. However, the framework does not take the economic perspective into account.

In addition to the literature on the methodologies used for evaluating eCare services or SCPs, previous work on the impact of the integration and adoption of SCPs is also available.

Bossen et al [9] conclude that integrating SCPs in the home environment of care receivers can facilitate and augment the current home care processes and enhance the cooperation between the several involved actors even more. Although larger pilot tests are needed to further evaluate the CareCoor system, initial tests revealed promising results and positive impacts for the care network.

In contrast with the results of Bossen et al, findings from the Whole Systems Demonstrator cluster randomized trials indicate that the effects of “second-generation” telecare are very limited and even without significant impact [18-20]. Except for a small benefit on psychological outcomes, the gain in quality of life is very small [18]. This also results in a very high cost-effectiveness ratio, meaning that the costs needed to obtain that small increase in quality of life are very high and far above the willingness to pay for it. According to Steventon et al [19] the telecare services as implemented in the Whole System Demonstrator do not lead to significant cost reductions in the use of care services.

Contradiction of the results of these researches indicates that more research is needed to clarify the impact of ICT-supported care service. This lack of evidence is seen as one of the barriers for adoption of eCare services [21]. The absence of the proof of positive effects also impacts the formulation of policies or new reimbursement systems [22]. This can affect the complete business model of the eCare service provider [10].

This paper identifies the expected impact of SCP integration for all actors involved. Via economic analyses, from the perspective of home care organizations, potential benefits and costs are compared with costs of current processes. Doing so, this research provides more clarity on viable economic business cases for SCPs.

## Methods

### Overview

**Figure 1:**
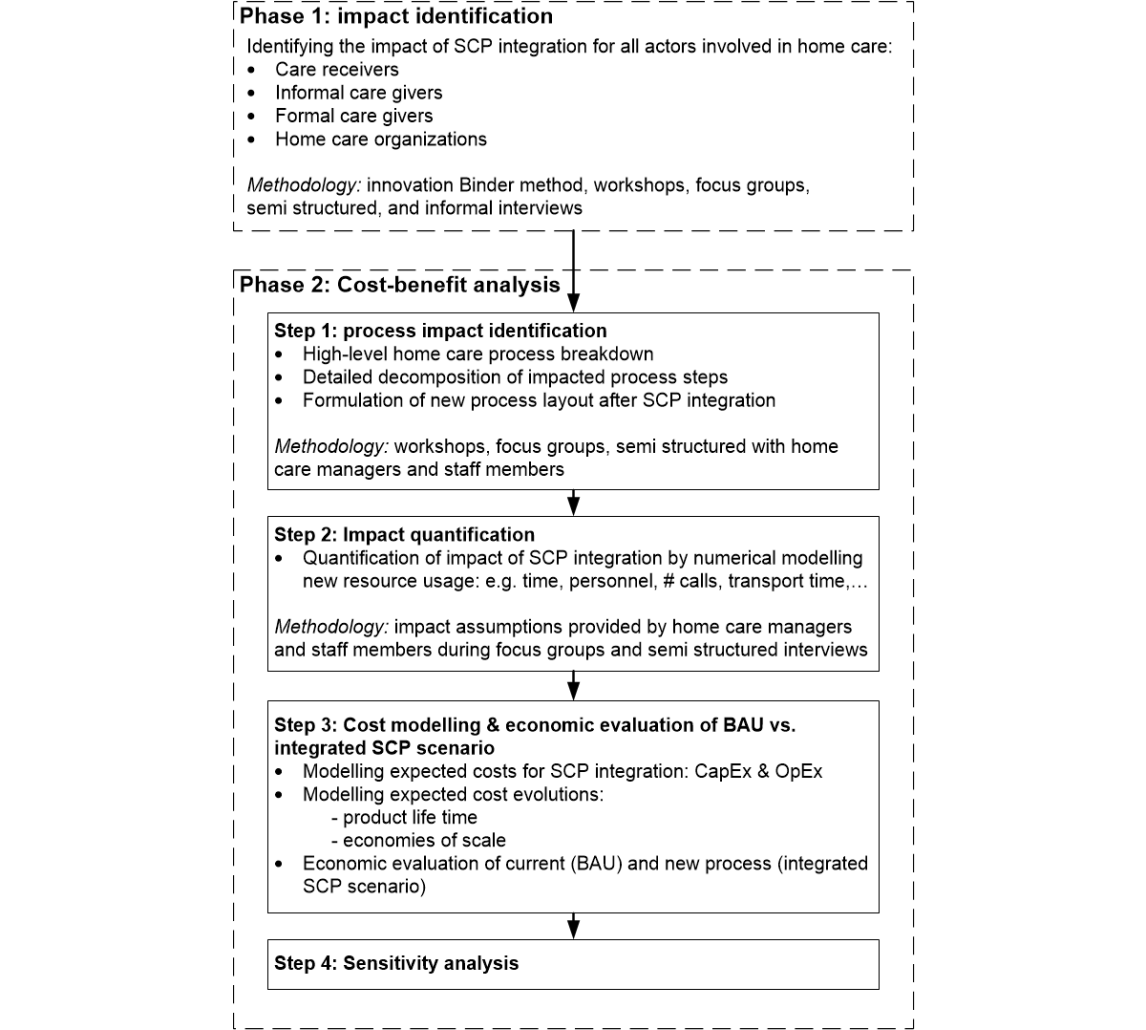
Schematic overview of the two-phase research method.

The methodology consists of 2 phases (see Figure 1). In the first phase, all various forms of potential impact and benefits are identified. During a second phase a 4-step economic cost-benefit analysis was modeled from the perspective of home care organizations.

### Phase 1: Impact Identification

First, expected fields of impact should be identified for each actor within the context of home care provisioning. The methodology known as the innovation Binder Approach [23] resulted after multiple iterations in input data from various perspectives such as technology, user or social, and business.

Additional input for this identification process resulted from workshops, focus groups, and semistructured and informal interviews with field experts such as managers and administrative staff members of home care organizations, home care providers, and technology providers. Both qualitative (eg, less anxiety, increased peace of mind, decreased burden of care) and quantitative impacts (eg, process excellence such as less administration or faster billing procedures) can be expected for the actors.

### Phase 2: Cost-Benefit Analysis From the Perspective of Home Care Organizations

#### Step 1: Identifying the Affected Home Care Processes via Process Breakdown Analyses

Adopting SCPs will affect several processes needed to provide home care such as administration tasks, communication, and sharing information. Via a high-level home care process breakdown, home care organizations were able to locate the most resource-intensive processes that could be affected after integrating SCPs. After this step, the identified process steps, care scheduling and billing processes in this case, were further decomposed.

#### Step 2: Quantification of Costs of the Current Business as Usual and Integrated Smart Care Platform Scenario

Effects such as better scheduling and task coordination have direct quantitative impacts in terms of monetary or time savings. In this project, no qualitative or quantitative research has been carried out on the impact on health utility for care receivers such as surveying the quality of life. Therefore, this work focuses on the changes in the care scheduling and billing processes of a care organization (direct quantitative benefits).

To do so, first the annual expense of the BAU was quantified. After SCP integration, the BAU could be affected, resulting in new costs. This assumed impact, provided by home care managers and staff members during focus groups and semistructured interviews, is modeled in as well. The difference or delta between the 2 scenarios is defined as a direct benefit if the costs of the integrated SCP scenario are lower than the costs of the BAU scenario.

#### Step 3: Economic Evaluation: Comparing the Integrated Smart Care Platform Scenario With the Business as Usual Scenario

The goal is to research whereas the resulting benefits justify all the operational costs and investments that are needed for adopting SCPs. Thus, after quantifying the expected effects, the BAU is compared with the new “Integrated SCP scenario.” Therefore, the costs of SCP integration are also modeled.

#### Step 4: Dealing With Uncertainty via Sensitivity Analyses

Although this cost-benefit model is developed with realistic data provided by service providers and experts from home care organizations, it is likely that variations of the values will occur. Therefore, we need to check whether the model still behaves normally with varying input values. Sensitivity analysis also provides us with a confidence interval for the result based on the input parameters modeled with known variations.

## Results

### Phase 1: Overview of Potential Impact Per Actor

In the first step of this research, the potential impacts or added values, resulting from the adoption of SCPs, are identified per actor involved. Methodologies used to identify the impacts are: the “Innovation Binder Approach,” as described in [23], informal and semistructured interviews with managers of care organizations, informal and professional care providers and care receivers.

Table 1 presents the various expected added values identified per actor along with the nature of impact (qualitative or quantitative). Within the context of home care, the following actors are included: (1) care recipient or patient, (2) informal care giver, (3) formal or professional care giver and home care organization, (4) care insurers or payers and society, (5) primary care, and (6) secondary and tertiary care.

**Table 1:** Identification of added values that can be expected per actor.

Actor	Added value description	Impact type: qualitative or quantitative
Care receiver	Control of the organization of care	Qualitative
	Strengthened involvement and empowerment	Qualitative
	Higher quality of care	Qualitative
	Higher state of peace of mind	Qualitative
	Higher state of self-management, less care dependent	Qualitative
	Lowered barriers for social contact and decrease of social isolation	Qualitative
	Better informed of existing and practical care support services	Qualitative
Informal care giver	Better care task coordination	Qualitative
	Improved quality of care or work atmosphere	Qualitative
	Less stress, less unexpected tasks, increased state of peace of mind	Qualitative
	Being better (and real time) informed	Qualitative
Formal care giver and care organization	Better care task coordination	Qualitative
	Improved quality of care or work atmosphere	Qualitative
	Less stress, less unexpected tasks, increased state of peace of mind	Qualitative
	Significant decrease in administration time (scheduling, adapting schedules, billing, etc)	Quantitative
	Reassuring care receivers when delay during care visits	Qualitative
Primary care (GPs)	Access to more complete care and context data	Qualitative
	Improved quality of care, faster and more complete diagnoses	Qualitative
	Being better (and real time) informed	Qualitative
Secondary and tertiary care	Access to more complete care and context data	Qualitative
	Being better informed	Qualitative
	Improved quality of care, faster and more complete diagnose	Qualitative
Care insurer or payer and society	More opportunities for prevention	Qualitative
	Savings because of delayed transition to care home	Quantitative
	Increase in cost-efficiency	Quantitative
	Overall higher quality of care	Qualitative
	Transition from curative to preventive care	Qualitative

Although the potential impact for every care giver is considerable, in what follows only the impact for the care organization is quantified. This actor is considered as an SCP initiator for 2 reasons:

- Several home care organizations already provide monitoring services such as personal alarm system and work with call centers. Offering SCPs toward their clients would extend the current service offers.
- SCPs have the potential to simplify and decrease the costs for organizing home care. Therefore, home care organizations have a potential incentive to adopt SCPs.

### Phase 2: Cost-Benefit Analysis From the Perspective of Home Care Organizations

The home care organizations themselves are convinced that a lot of improvement is possible in the process of home care provisioning. In order to detect which process steps would be affected by SCPs, semistructured interviews and focus groups with care organizations were carried out to collect data to be able to quantify the current costs for billing and rescheduling processes.

These data served as input for a numerical model to calculate the potential benefits and costs. In what follows, all results of the 4-step model (Figure 1) are discussed.

#### Step 1: Process Impact Identification

In the first step, the complete process of home care provision is broken down into several main and sub process blocks. This allowed the managers and staff members of the home care organization to locate process steps that potentially would be affected when integrating SCPs.

**Figure 2:**
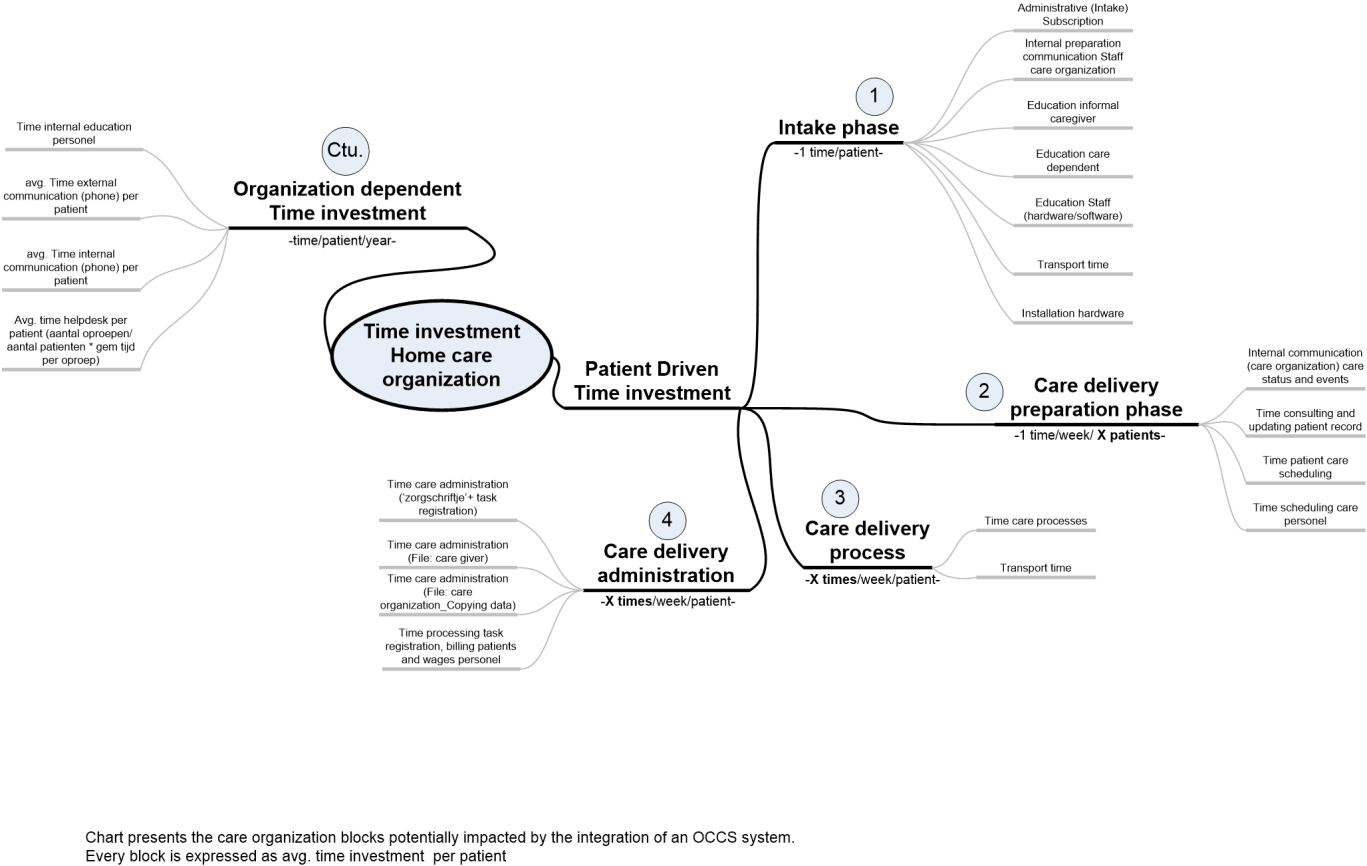
High-level process breakdown of home care delivery.

**Figure 3:**
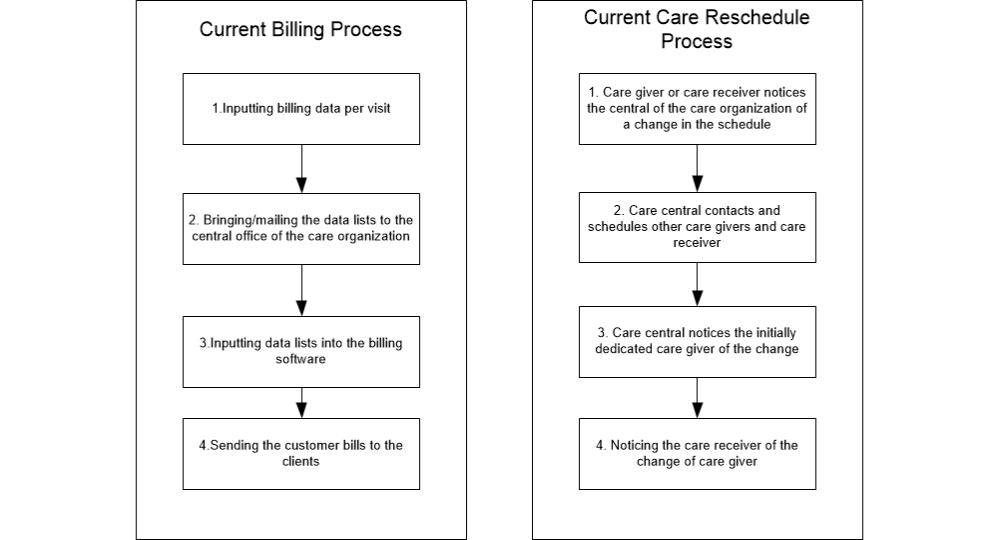
Process decomposition of current billing and care rescheduling processes—business as usual (BAU) scenario.

Figure 2 presents the high *-* level process spider chart for home care provisioning. The main process blocks for home care provisioning are patient intake phase, preparation of the care delivery, actual care delivery, and care delivery administration. Two-process steps were identified by the expert team as potentially impacted by adopting SCP. First, the current process for billing for home care was identified and second, the process that takes place when something has to change to the actual care schedule. For instance, when a caregiver gets sick, all planned appointments need to be reallocated to other care givers. A second example provided by the expert group is: when a client visits the hospital, all planned care visits should be replaced with others, otherwise the care givers would have no work. A more detailed decomposition of both processes is shown in Figure 3.

#### Step 2: Quantification of Costs of the Current Business as Usual and Integrated Smart Care Platform Scenario

##### The Process Break Down and Resource Usage of the Current Business as Usual Scenario

In the next step each process block of the current billing process is quantified in terms of cost per year. The same is done for the rescheduling process. Relevant data in order to calculate the cost of the current processes or business as usual are presented in Table 2.

**Table 2:** Cost parameters and drivers used to calculate the cost of the business as usual (BAU) process.

Numerical parameters for the current billing process	Numerical parameters for the current rescheduling process
Number of care visits per month	Frequency of care rescheduling in terms of percentage of the total amount of planned care visits
Total amount of care givers	Telecommunication costs for calling the central administration office
Full-time equivalents (FTEs) of care providers	Average time needed to make the rescheduling exercise (not every care provider can be reallocated to a changed care visit due to professional or personal reasons (eg, care provider must speak Dutch, cannot be pregnant because of potential diseases of the cat of the care receiver)
Time needed to input the data into the back-end system	Time needed to inform the original dedicated care giver
Cost for mailing the monthly visit records of the care giver to the care organization	Scheduled visits per month
Time needed for inputting the data after each visit	Number of rescheduled visits per month
Average wages of the administration staff and the care providers	
Transport time	
Transport frequency	
Time needed for rework due to errors	

**Figure 4:**
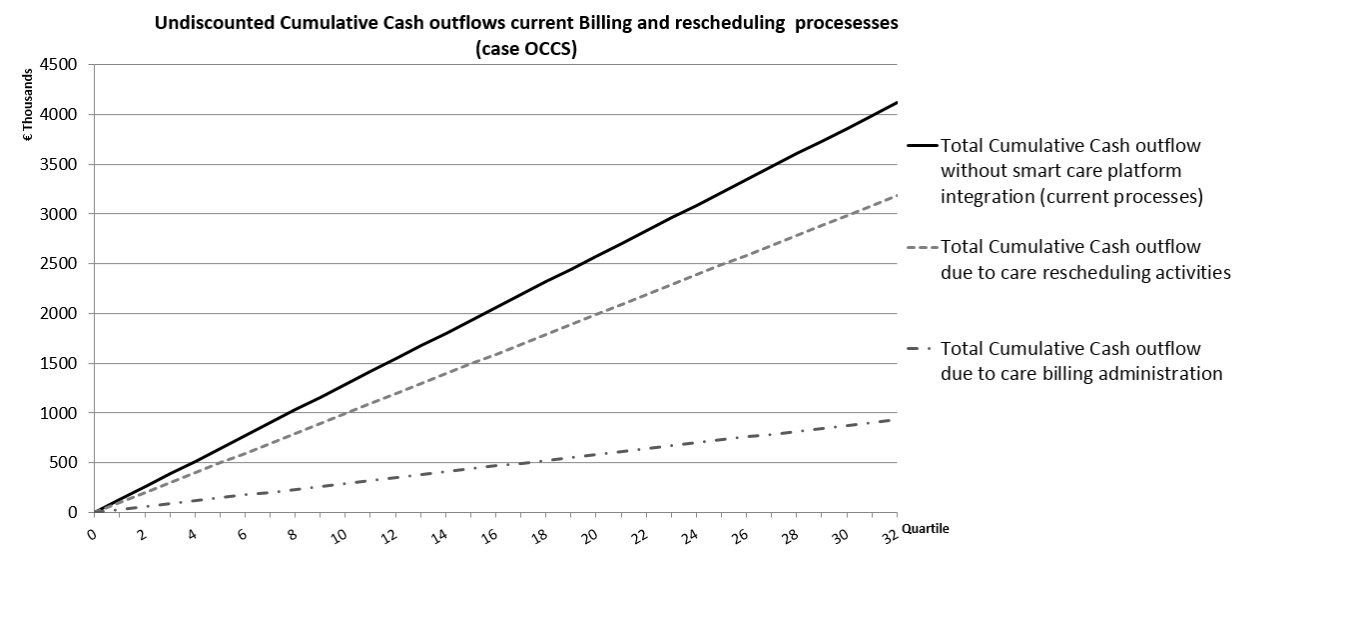
The costs for the current rescheduling activities are more than 3 times higher than the current costs for billing administration. This is mainly caused by the wages of central office staff members who do the actual rescheduling (see Multimedia Appendix 1).

The model was initially designed for an East Flemish Care organization involved in the OCCS project, but is not limited to this organization. This region counts about 881 full-time equivalent home care givers who are members of the care organization. All data and results are valid within the scope of the OCCS project [12]. According to the managers of the care organizations, the input provided and process issues described are similar for all Flemish and even Belgian care organizations. For detailed data of current billing and rescheduling processes see Multimedia Appendix 1.

Figure 4 presents the current cumulative cash outflows per quartile for both the billing and rescheduling processes. In total, these 2 processes cost about €510,000 per year for this provincial division of the Flemish care organization involved in the OCCS project.

##### The Process Break Down and Resource Usage for the Integrated Smart Care Platform Scenario

**Figure 5:**
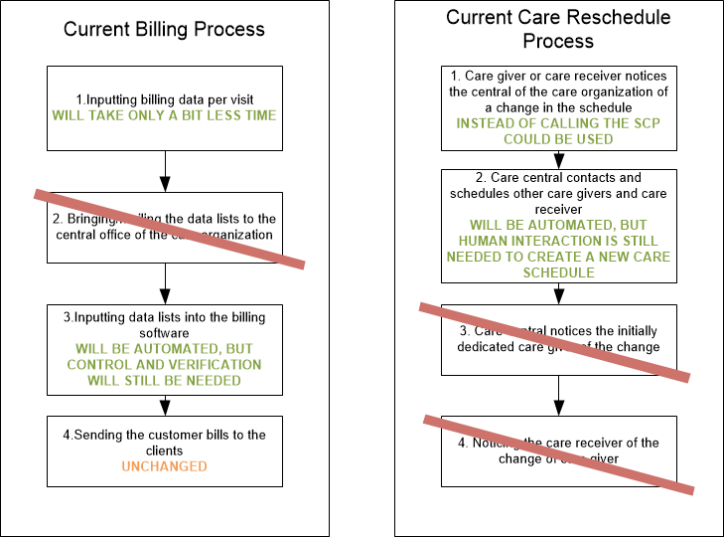
Process decomposition of new billing and care rescheduling processes (adaptations are indicated in green)—integrated smart care platform (SCP) scenario.

Together with the care organization we modeled how an SCP would affect the current billing and rescheduling processes. Some process steps would remain unchanged; others would even disappear or would be affected. Figure 5 shows what process steps would be affected and how.

**Figure 6:**
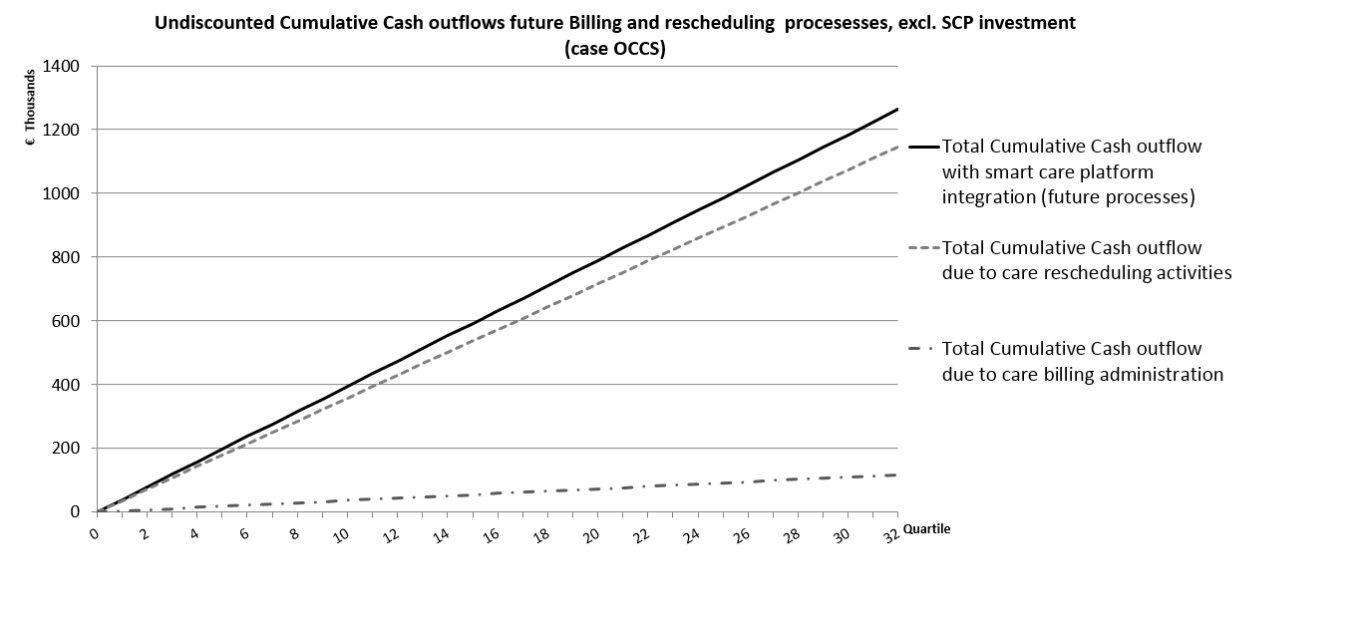
In the new integrated smart care platform scenario, the billing process is almost completely automated. That explains the low cumulative cash outflow due to the future billing processes (see Multimedia Appendix 2).

For detailed data on the affected process parameters, see Multimedia Appendix 2. Figure 6 shows the expected cumulative cash outflows of the new processes.

**Figure 7:**
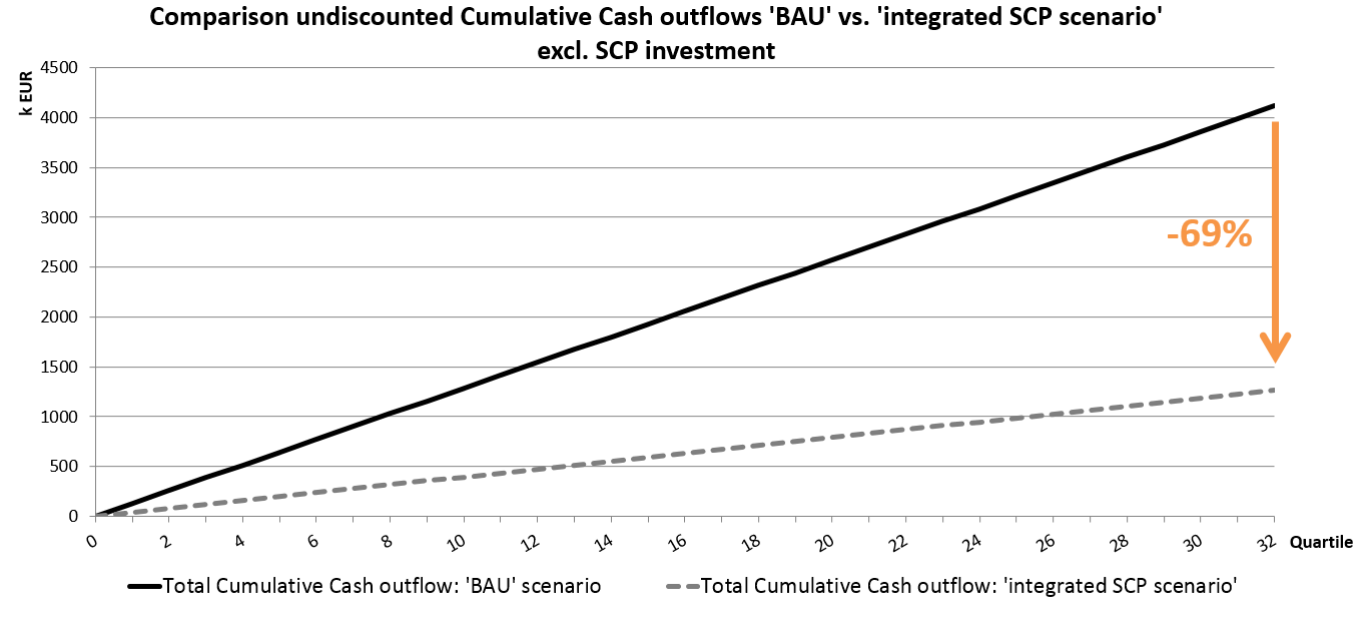
Automating the billing processes and rescheduling processes would lead to a process cost reduction of 69%.

Given the validated impact assumptions such as reduced time needed for putting in the billing information, fewer telephone calls, no correspondence needed anymore, and so on, the total annual expense of the new processes, investments in SCP excluded would decrease to €160,000 per year. This means a reduction of 69% of the total cost of the current billing and rescheduling processes can be obtained. Figure 7 presents the comparison between the cumulative expected costs of the current and future billing and rescheduling processes.

A clear difference between the costs of the current and potential new billing and care rescheduling processes can be seen. But the latter requires a significant investment in order to reach these potential savings. Furthermore, it is expected that the data inputting process could be more time-efficient for the care provider by the use of the smart care app on the mobile phone. For the provincial home care organization involved in the OCCS project, this could free up nearly 11,000 h per year ([1488 min/year − 744 min/year] × 881 FTEs); see Multimedia Appendices 1 and for data. This time could be spent with the care receiver, resulting in better quality of care (more quality time for the patient) without affecting the cost.

**Table 3:** Investments to integrate O’CareCloudS (OCCS), based on expert estimations within OCCS and sector averages.

Description of investment	Value	Unit
Every care provider needs a (basic) mobile phone, not only the people who work full-time, but also the people who work part time. (The lifetime of these devices is currently set at 3 years. Then they need to be replaced) *[CapEx]*	80	€/care provider
Every care provider needs a mobile telecommunication subscription. (There exist special group tariffs for care organization, that is why this annual expense is initially modeled rather low) *[OpEx]*	40	€/year per care provider
Each care provider needs to have access to OCCS. An annual subscription cost is modeled per care provider. *[OpEx]*	20	€/year per care provider
Each care provider needs to be educated to understand the functionalities of the SCP (2 h of education) *[CapEx]*	31	€/care provider
The SCP needs to be integrated into the back-end systems (1 FTE during 3 months) *[CapEx]*	14,700	€
An annual operational cost which is modeled as a percentage of the integration cost is needed to keep the SCP up and running *[OpEx]*	5%	

#### Step 3: Investment Modeling and Economic Evaluation

##### Smart Care Platform Investment Modeling

The expected savings can only be obtained if the home care organization invests in an SCP system like OCCS. These investments are modeled in Table 3.

Furthermore, economies of scale are modeled for the SCP subscription cost per care provider. This is modeled as a staircase function, driven by the number of care providers connected with the SCP.

The rollout of an SCP within the complete care organization is modeled as a staircase function as well. This was asked by the managers of the home care organization. Each quartile, 25% of all care givers are provided with the needed hardware and the education time. After 1 year, all care givers are connected with the SCP.

##### Economic Evaluation: Comparing the Integrated Smart Care Platform Scenario With the Business as Usual Scenario

Now that the impact and costs of SCP integration are known, we can investigate whether the impact is still positive after taking into account all the costs for SCP deployment.

**Figure 8:**
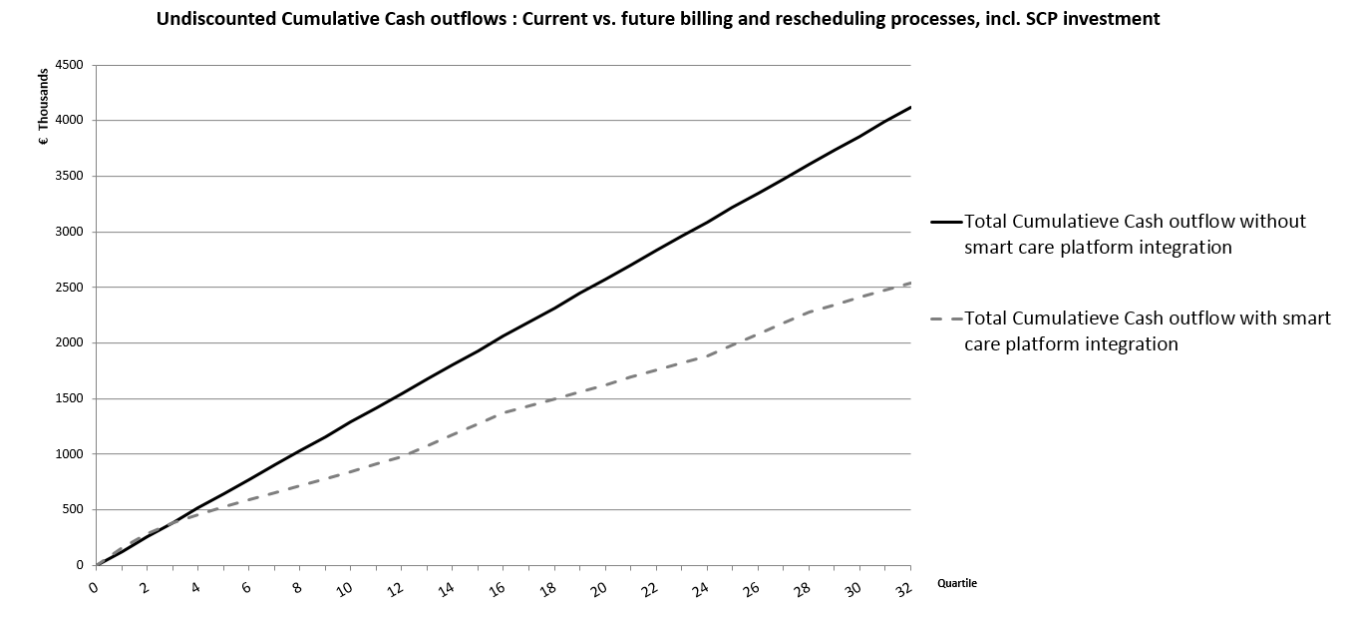
In the first year, the cash outflow of the integrated smart care platform (SCP) scenario is the same as for the current business as usual (BAU) scenario because of the initial investments. But after that, one can see clearly the potential savings of integrating an SCP.

The following graph (Figure 8) shows the expected evolution of the undiscounted cash outflow in a situation in which a smart care system would be deployed in 1 year compared with the costs of current billing and rescheduling processes.

Based on the provided data, integrating an SCP would have a payback time which is less than 1 year. Within a period of 8 years after the investment, a total cost reduction of 38% can be expected.

**Figure 9:**
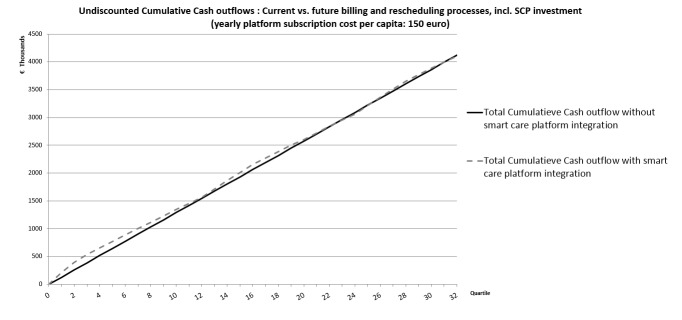
Expected evolution of the cumulative cash outflow in case the annual cost per care provider would be € 150. This is the upper boundary for the yearly costs per care provider.

From Figure 9 one can see that, according to this model, the total annual expense per care provider can maximum increase to about €150 per person per year. At that level, the expected costs of the SCP integration would be the same as the current costs, everything smaller than €150 would lead to savings.

**Table 4:** Modeled distributions for uncertain input parameters, based on expert estimations within O’CareCloudS (OCCS).

Parameter	Modeled distribution
Number of hours needed for education (h)	Normal distribution with parameters mean=2.00, SD=0.32
Annual SCP maintenance costs (% of integration cost)	Normal distribution with parameters mean=0.05, SD=0.01
SCP back-end integration cost (€)	Lognormal distribution with parameters location=104,000, mean=14,700, SD=3498.6
Cost for mobile phone (€)	Maximum extreme distribution with parameters likeliest=80, scale=1.94
Yearly Telco subscription for the care provider (€/year)	Normal distribution with parameters mean=40, SD=11.76
Yearly smart care platform subscription cost for the care providers (€/year)	Beta distribution with parameters minimum=15, maximum=100 alpha=1.2, beta=2.6

#### Step 4: Dealing With Uncertainty

To take uncertainties into account, such as the assumed impacts on both affected processes, a sensitivity analysis is performed. Table 4 depicts the variations on uncertain input parameters.

**Figure 10:**
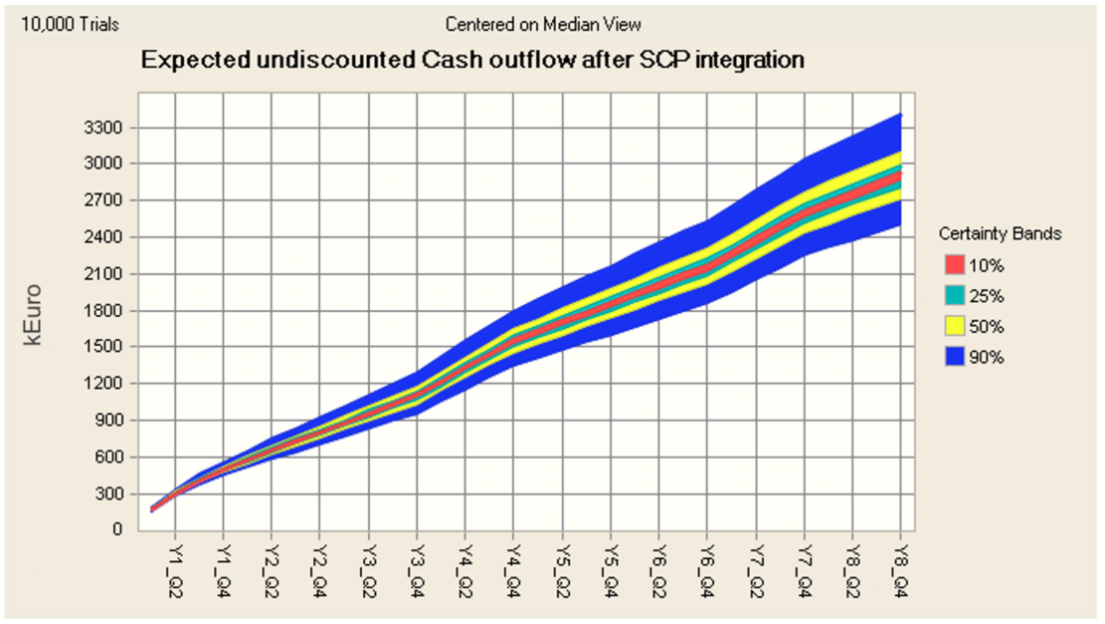
Expected undiscounted cumulative cash outflow with CIs 90%, 50%, 25%, and 10%. In the worst-case scenario, the cost of the billing and rescheduling process will still cost 18% less than in the current situation.

The result of the sensitivity analysis indicates a 90% chance that within a period of 8 years after the investment in an SCP, the cumulative undiscounted cash outflow will lie between k€2400 and k€3400 (see Figure 10).

**Figure 11:**
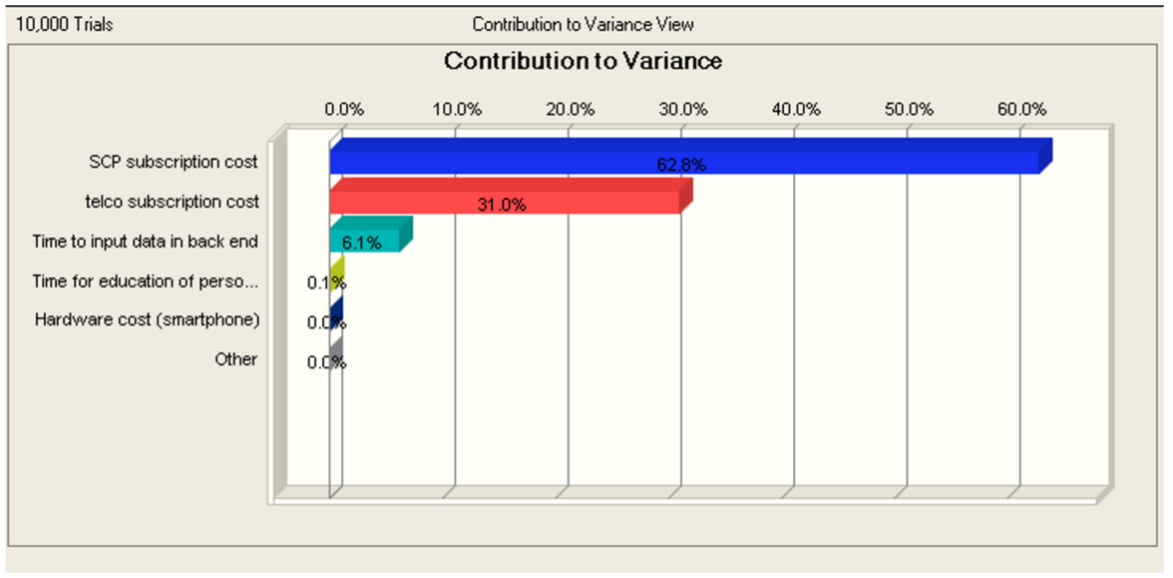
The annual subscription cost for smart care platforms is expected to have the biggest impact on the expected savings, followed by the annual expenses for telecommunication.

Testing the model robustness indicates that the SCP subscription cost is the driving parameter in this model (see Figure 11). This is acceptable as the variance on this parameter is rather high and because of the annual effect of it. The same is true for the Telco subscription cost. This means that it will be important to negotiate good subscription prices for both access to the SCP and for the telecommunication subscriptions.

## Discussion

### Principal Findings

Integrating SCPs such as OCCS could affect the care administration process of care organizations. Based on the provided process data of BAU and “integrated SCP scenario,” an annual cost reduction of 37-38% could be expected. This cost reduction does not result from the SCP’s main purpose, being sharing care data or monitoring care receivers, but from the fact that digitizing one or more parts of an often time-intensive manual process can save expensive resources.

The results indicate that at least for care organizations, which are often important actors in the home care provisioning for elderly, the impact of integrating SCPs within their own scheduling and billing software is positive. This is important because literature indicates that the impact of SCPs on the quality of life of care receivers is rather limited and still not convincing enough to drive a viable adoption.

This SCP integration would not require involvement of the care receivers or their informal caregiver. Only professional care givers within the organizations could consult the care information. Initially, for the care receivers and informal care givers the added value of such a system would remain very limited.

Therefore, we believe that until the added value of SCPs for the care receiver increases to a critical level for which there exists a viable willingness to pay, the adoption of SCPs will be driven by a positive affected actor such as the care organizations. This could be a first step to digital integration and collaboration of care organizations and a first step toward a patient-centred care system.

Once all personnel of the care organization receive education and familiarize themselves with the functionalities, the home care organization can open the other functionalities of the SCPs also toward the care receivers, their informal care givers, and other care providers. In this way also other actors with a lower willingness to pay, because of the less direct quantitative benefits of SCPs, can experience the added values of SCPs.

As this research is a part of the Flemish OCCS project, the results are pertaining to the Flemish homecare organization involved in the project. As the field experts who provided data for this research stated that the situation is the same in the entire Belgium and is the same even in many Western European countries, therefore, the findings could be generalized.

### Limitations

Although there are many beneficial impacts due to SCP integration, it should be noted that SCP adoption will result in some challenges and threats. Often there are concerns about privacy, data ownership, and replacing human care toward automated less personal care. It was not the focus of this research to describe all potential barriers. The results of the economic evaluation should not be affected by taking these challenges and threats into account.

Another point of remark is the single-sided perspective of the economic evaluation. Considering the case of the home care organizations alone is a well-considered choice because we strongly believe that these actors will drive adoption for SCPs. However, other actors such as society, care payer, and formal care providers could also experience economic impacts. From that point of view, the results of the analyses are probably an underestimation of the real effects. Future research on the evaluation from more perspectives complemented with a study on the impact on quality of life can bring more clarity.

### Conclusions

This work envisions to identify, describe, quantify, and evaluate the impact of integrating new “Cloud-like” smart care platforms into the current home care processes. The goal of these platforms is to offer trusted information and knowledge-based services related to the care organization and delivery to the client or patient. These services aim to support and foster communication on the daily care-related needs, the social needs, and daily life assistance.

One of the goals of integrating SCPs is to foster open communication and data sharing among all the involved actors (eg, care organization, general practitioner, formal and informal care givers). Thus, in order to stimulate usage of SCPs, all actors involved should benefit from it or at least not be affected negatively.

The research indicates that all actors could benefit from the integration of SCPs. Care receivers can expect a higher quality of life, informal care givers could face a higher state of peace of mind, and formal care providers can provide the same quality of care while there could be more quality time available. Care organizations can optimize their care administration processes and push the level of digitization even further. Finally, care insurers and society in general could profit because of the possibility to provide personalized prevention and decrease or postpone the move to care homes and let the elderly stay at home instead. Although these expected effects sound acceptable, it is not clear yet whether these impacts will convince care receivers to adopt SCPs.

However, when we step away from the main goals of integrating SCPs and focus on the potential effects that result from digitizing and optimizing the current administration of home care processes (billing and care scheduling in particular), our quantification model indicates that a cost reduction for the home care organization of 37-38% could be expected and thousands of hours per year could be freed up for providing quality care by optimizing the current administrative tasks. Thus, if SCPs could be integrated within the already existing back-end systems of care organizations or vice versa, the savings potential could be a viable driver for the adoption of SCPs by home care organizations.

## Appendix

Multimedia Appendix 1 Current process breakdown (business as usual scenario).

Multimedia Appendix 2 Future process breakdown (integrated smart care platform scenario).

## Abbreviations

BAU: business as usual
FTE: full time equivalent
OCCS: O’CareCloudS
SCP: smart care platform
