# Supplementary material for: Evaluating the Economic Impact of Smart Care Platforms: Qualitative and Quantitative Results of a Case Study
Source: JMIR Med Inform. 2016 Oct 31;4(4):e33. doi: 10.2196/medinform.5012 (PMC5108925; doi:10.2196/medinform.5012)
Supplement: Multimedia Appendix 1 [file medinform_v4i4e33_app1.pdf]

Multimedia Appendix 1: Current process breakdown

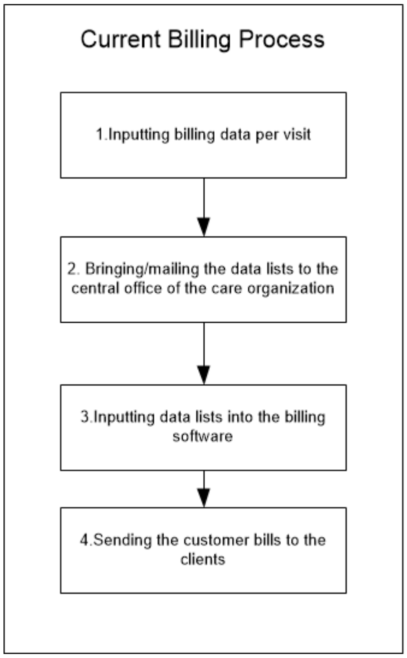

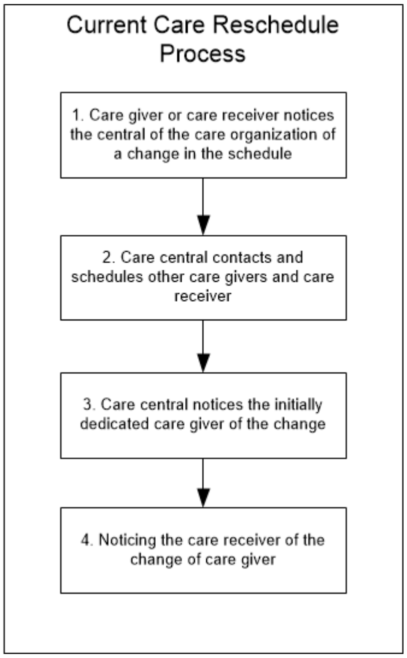

In following two tables a concise overview of the data is presented. All data was validated by experts involved in the OCCS project.

Table 1: Overview of the data for the current billing process

Description data parameter	*Value*	*Unit*
Time needed for inputting data per visit	2	min/visit
# visits per month per FTE	62	visits/month
Frequency of data list delivery by the care provider to the care organization	12	deliveries/year
Cost of envelopes to send the lists	12	euro/year
Time needed for inputting one line of the data list into the back end system	0.31	Min/data line
# care givers in ‘Interregio Gent’ (full time + part time)	1719	Persons
Full Time equivalent of the total amount of care givers	881	FTEs
Total amount of data lines inputted in the backend system by the central administration (March 2014)	88000	data lines/month
% rework due to mistakes in inputting	4%	of # data

Table 2: Overview of the data for the current rescheduling process

Description data parameter	*Value*	*Unit*
Time needed by the care receiver or care provider to inform the care central	1.5	min/call
Telco costs needed to inform the care central (in case the care provider calls with own mobile)	0.5	euro/call
chance that that a visit needs to be rescheduled	15%	Of planned
# visits per month per FTE	62	visits/month
Chance that it the care receiver informs the care central him/herself	70%	
Time needed to inform other care provider	1	min/contacted care provider
Costs Telco for the care administration	0.01	euro/min
Average amount of care actors to contact (number of calls to make)	4	

time needed to make the new care schedule	4	Min

Based on these inputs, the model provides following results on current resource usages:

Table 3: Resource usages for the current billing process

Description data parameter	*Value*	*unit*
Total time needed per FTE when inputting billing data when he is with the care receiver (time is paid by care receiver)	1488	min/year
Total time needed for the care administration to put in all the billing data of the care givers into the back end system	339120	min/year
Costs for the care administration to put in all the billing data of the care givers into the back end system	96084	euro/year
Costs to provide each care provider with 12 envelopes to send the data lists once a month	20628	euro/year
Total cost for the care organization caused by the current billing process	116712	euro/year

Table 4: Resource usages for the current care rescheduling process

Description data parameter	*Value*	*unit*
Total time needed per care provider to contact and discuss the new care schedule with the permanency station (central office)	496	min/year
Total cost of wages for the care organization to pay for the time needed of each care provider to contact and discuss the new care schedule with the permanency station	119180	euro/year
Total costs for compensating the telecommunication cost of the care providers when they called with their own device to the care central	14748	euro/year
Total telecommunication costs for the permanency station due to calling to the care providers (central office)	6292	euro/year
Total cost for the wages of the people of the permanency station (central office)	264644	euro/year
Total cost for the care organization caused by the current care rescheduling process	397981	euro/year

Table 5: Total cost of current billing and care rescheduling processes

Total cost for the care organization caused by the current billing process	116712	euro/year
Total cost for the care organization caused by the current care rescheduling process	397981	euro/year
Total cost of the current billing and rescheduling processes	514693	euro/year

Next to high costs for the processes we notice from the table 13 that all the clients in total pay for about 22000 hours (1488 min/year per FTE x 881 FTEs) per year for filling in the billing data.
