# Supplementary material for: Evaluating the Economic Impact of Smart Care Platforms: Qualitative and Quantitative Results of a Case Study
Source: JMIR Med Inform. 2016 Oct 31;4(4):e33. doi: 10.2196/medinform.5012 (PMC5108925; doi:10.2196/medinform.5012)
Supplement: Multimedia Appendix 2 [file medinform_v4i4e33_app2.pdf]

Multimedia Appendix 2: Future process breakdown

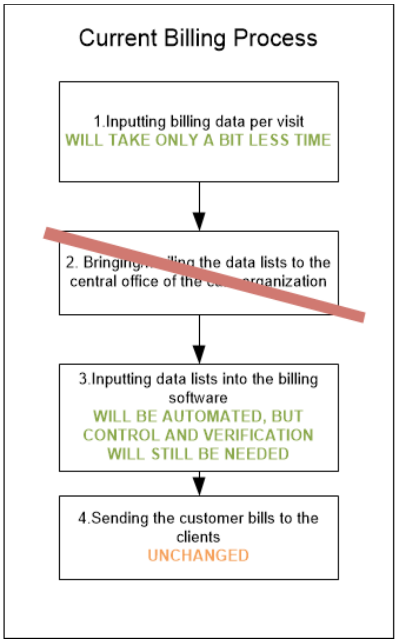

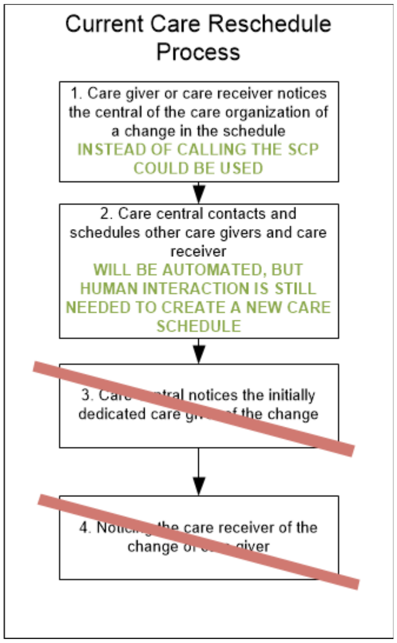

Following data of the new processes was validated by field experts involved in the research project.

Table 1: Input for the Billing process when a smart care platform is integrated

Description data parameter	*Value*	*Unit*
Time needed for inputting data per visit	1	min/visit
# visits per month per FTE	62	visits/month
Frequency of data list delivery by the care provider to the care organization administration	0	deliveries/year
Cost of envelopes to send the lists	0	euro/year
Time needed for inputting one line of the data list into the back end system	0.05	Min/data line
# care givers in 'Interregio Gent' (full time + part time)	1719	persons
Full Time equivalent of the total amount of care givers	881	FTEs
Total amount of data lines inputted in the backend system by the central administration (March 2014)	88000	data lines/month
% rework due to mistakes in inputting	4%	of # data lines/month

Table 2: Input for the Rescheduling process when a smart care platform is integrated

Description data parameter	*Value*	*unit*
Time needed by the care receiver to inform the care central	1.5	min/call
Time needed by the care provider to inform the care central	0.33	min
Telco costs needed to inform the care central (in case the care provider calls with own mobile)	0	euro/call
chance that that a visit needs to be rescheduled	15%	Of planned monthly visits
# visits per month per FTE	62	visits/month
Chance that it the care receiver informs the care central him/herself	70%	

Time needed to inform other care provider	0	min/contacted care provider
Costs Telco for the care administration	0.01	euro/min
Average amount of care actors to contact (number of calls to make)	4	
time needed to make the new care schedule	4	min

The new process would lead to following resource usages exclusive the investment in a smart care platform!

Table 3: Resource usages for the billing process when a smart care platform is integrated

Description data parameter	*Value*	*unit*
Total time needed per FTE when inputting billing data when he is with the care receiver (time is paid by care receiver)	744	min/year
Total time needed for the care administration to put in all the billing data of the care givers into the back end system	51859	min/year
Costs for the care administration to put in all the billing data of the care givers into the back end system	14693	euro/year
Costs to provide each care provider with 12 envelopes to send the data lists once a month	0	euro/year
Total cost for the care organization caused by the current billing process	14693	euro/year

Table 4: Resource usages for the rescheduling process when a smart care platform is integrated

Description data parameter	*Value*	*unit*
Total time needed per care provider to contact and discuss the new care schedule with the permanency station (central office)	11	min/year
Total cost of wages for the care organization to pay for the time needed of each care provider to contact and discuss the new care schedule with the permanency station (central office)	2498	euro/year
Total costs for compensating the telecommunication cost of the care providers when they called with their own device to the care central (now included in investment for mobile subscription)	0	euro/year
Total telecommunication costs for the permanency station due to calling to the care providers(central office) (now included in investment for mobile subscription)	0	euro/year
Total cost for the wages of the people of the permanency station (central office)	140679	euro/year
Total cost for the care organization caused by the current care rescheduling process	143177	euro/year

Table 5: Total cost of billing and care rescheduling processes when a smart care platform is integrated

Total cost for the care organization caused by the future billing process	14693	euro/year
Total cost for the care organization caused by the future care rescheduling process	143177	euro/year
Total cost of the future billing and rescheduling processes	157870	euro/year
